# Microbe observation and cultivation array (MOCA) for cultivating and analyzing environmental microbiota

**DOI:** 10.1186/2049-2618-1-4

**Published:** 2013-01-09

**Authors:** Weimin Gao, Dena Navarroli, Jared Naimark, Weiwen Zhang, Shih-hui Chao, Deirdre R Meldrum

**Affiliations:** 1 Arizona State University, Tempe, AZ 85287, USA

**Keywords:** Microbe observation and cultivation array (MOCA), Cultivation, Growth, Microbiota

## Abstract

**Background:**

The use of culture-independent nucleic acid techniques, such as ribosomal RNA gene cloning library analysis, has unveiled the tremendous microbial diversity that exists in natural environments. In sharp contrast to this great achievement is the current difficulty in cultivating the majority of bacterial species or phylotypes revealed by molecular approaches. Although recent new technologies such as metagenomics and metatranscriptomics can provide more functionality information about the microbial communities, it is still important to develop the capacity to isolate and cultivate individual microbial species or strains in order to gain a better understanding of microbial physiology and to apply isolates for various biotechnological applications.

**Results:**

We have developed a new system to cultivate bacteria in an array of droplets. The key component of the system is the microbe observation and cultivation array (MOCA), which consists of a Petri dish that contains an array of droplets as cultivation chambers. MOCA exploits the dominance of surface tension in small amounts of liquid to spontaneously trap cells in well-defined droplets on hydrophilic patterns. During cultivation, the growth of the bacterial cells across the droplet array can be monitored using an automated microscope, which can produce a real-time record of the growth. When bacterial cells grow to a visible microcolony level in the system, they can be transferred using a micropipette for further cultivation or analysis.

**Conclusions:**

MOCA is a flexible system that is easy to set up, and provides the sensitivity to monitor growth of single bacterial cells. It is a cost-efficient technical platform for bioassay screening and for cultivation and isolation of bacteria from natural environments.

## Background

During the past over two decades, the use of culture-independent nucleic acid techniques, represented by ribosomal RNA gene cloning library analysis, has unveiled the tremendous microbial diversity that exists in natural environments [1]. In sharp contrast to this great achievement is the current inability to cultivate the majority of bacterial species or phylotypes revealed by molecular approaches. One of the major difficulties for microbial ecology is that conventional cultivation methods provide access to only a very small fraction of the microbial diversity and more than 99% of naturally occurring microbes are considered ‘unculturable’ on standard culture media [2]. Although recent new technologies such as metagenomics and metatranscriptomics can provide more functionality information about microbial communities [2,3], it is still important to develop the capacity to isolate and cultivate individual microbial species or strains in order to gain better understanding of microbial physiology and to apply isolates for various biotechnological applications [4].

A new view is emerging among microbial ecologists that the majority of so-called ‘unculturable’ microbial species simply have not been cultured yet. In line with this view, more research is necessary to enhance the ability to culture microbes, in order to reduce the dependence on indirect and cumbersome metagenomic approaches [5,6]. Recent developments in improving traditional cultivation techniques have shown that some conventionally unculturable species can in fact be grown as pure cultures. Tamaki *et al.* reported that the use of gellan gum instead of agar as the solidifying agent could greatly improve the cultivability of novel microbes on solid media [7,8]. Kaeberlein *et al.* designed a diffusion chamber to grow previously uncultivated pure isolates of marine origin [9], and the same strategy was also successfully used in cultivation of groundwater microorganisms [10]. Stevenson *et al.* achieved similar results with soil microbes by fine-tuning the oxygen concentration and nutrient levels [11]. Knowledge obtained through metatranscriptome analysis has also been used in directed cultivation of bacteria [12]. These examples shown that many microbial species can be cultured as long as the environments are optimized for growth.

Recently, some non-traditional cell-isolation technologies have been introduced to isolate targeted cells for pure culture cultivation. For example, Huber *et al.* used optical tweezers to track and isolate an extremophilic archaeon from a microbial community in a terrestrial hydrothermal vent field [13]. This method of single-cell manipulation provides a new way to grow pure microbial cultures from single mother cells, but it has the disadvantage that the identification and manipulation of the bacteria is an extremely labor-intensive process. By contrast, a high-throughput isolation method has been presented using encapsulated single bacteria in droplets of gel [14], resulting in the successfully cultivation of pure cultures from marine microorganisms. Oligonucleotide probes were used to identify the species after isolation.

One of the remaining hurdles for bacterial cultivation is that fine-tuning the growth condition for any specific species is a daunting effort, especially with the widely diverse microbiota in the ocean and soil environments. Consequently, a high-throughput platform is urgently needed to perform trials of different cultivation conditions in parallel. Previously, several high-throughput microtiter-plate-based cultivation platforms have been developed for marine and aquatic water column bacteria, and these have contributed greatly to the successful cultivation of previously uncultivated bacteria [15,16]. Most recently, a chip-based version of a Petri dish and diffusion chamber has been developed to address the same purpose [17,18]. Microfluidic ‘lab-on-a-chip’ (LOC) devices have been used for co-cultivation of various bacterial strains and species [19,20]. These devices are complicated in structure, utility, and fabrication, and are therefore less useful for general microbiologists.

In this study, we developed a parallel cultivation set-up that incorporates streamlined processes and is compatible with downstream genomic analysis. It spontaneously isolates environmental bacteria into miniature incubation chambers from a mixed microbial community. The key component of the system is the microbe observation and cultivation array (MOCA), which uses a Petri dish that contains an array of droplets with an oil covering as cultivation chambers. During cultivation, the growth of bacteria across the droplet array can be monitored using an automated microscope, which can produce a real-time growth record. Compared with conventional cultivation methods, MOCA provides streamlined preparation, parallel cultivation, and real-time observation, and unlike other chip-based platforms [16-19], MOCA does not require complicated engineering techniques or equipment for fabrication. We have found that bacterial growth across the droplet array has a high level of uniformity when the initial cell density is more than 10 cells/μl, and thus MOCA provides a novel platform for bioassay screening. When the cell occupancy in droplets is at the single-cell level, real-time image recording can be used to monitor the growth and morphological development of microcolonies derived from single bacterial cells. The droplet culture developed from a single bacterial cell can be transferred using a micropipette [21] for bulk cultivation or further molecular analysis.

## Methods

### Microbe observation and cultivation array

The MOCA Petri dish is produced using a technique known as microscale plasma activated templating (μPLAT) [22,23]. The μPLAT method uses an inexpensive plasma-treatment process to create hydrophilic patterns on Petri dishes (60 mm diameter; Falcon; Becton Diskinson Labware, Franklin Lakes, NJ, USA); the air plasma makes the originally hydrophobic polystyrene surfaces of the Petri dishes hydrophilic.

**Figure 1:**
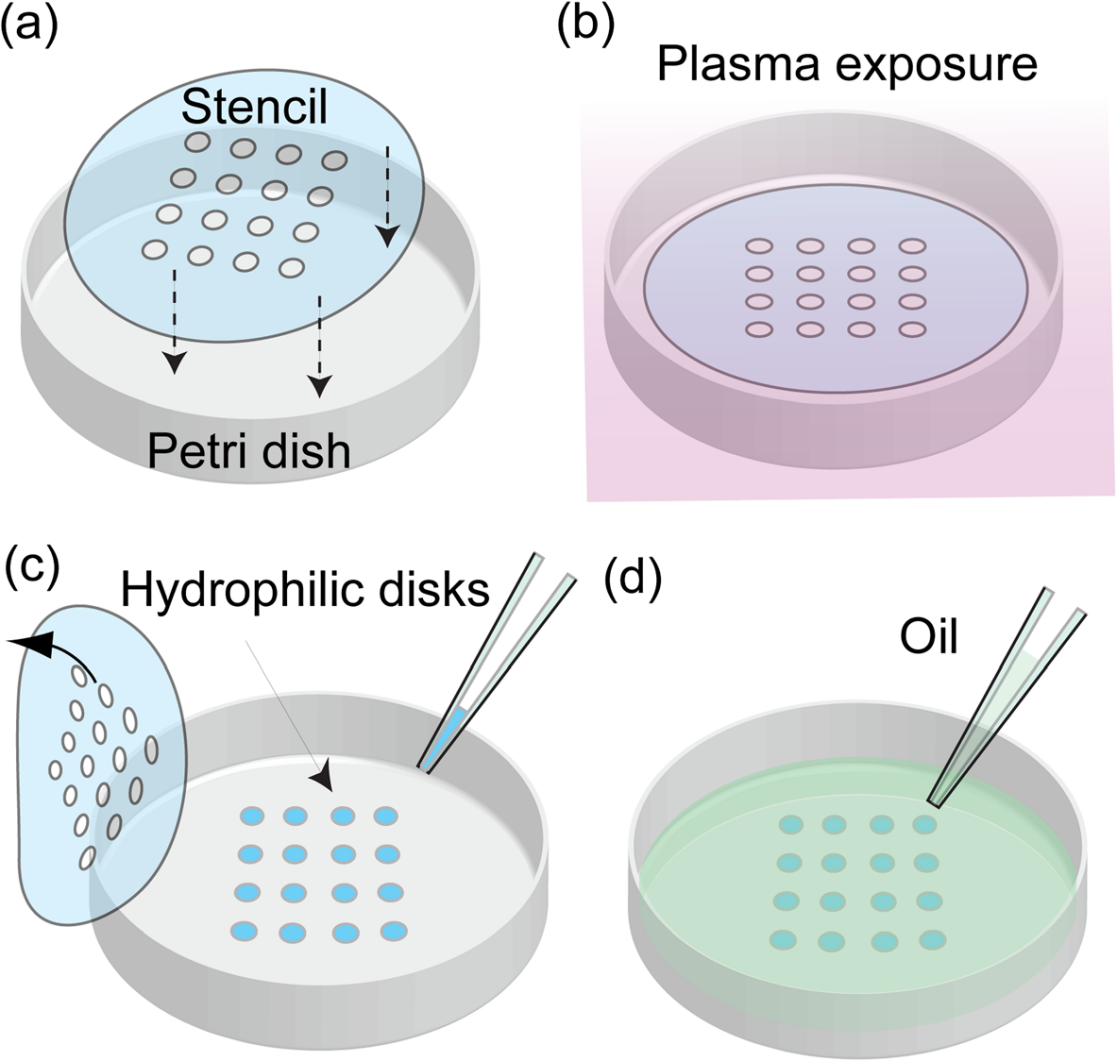
**Scheme of microbe observation and cultivation array (MOCA) droplet preparation.** (**a**) A stencil with an array of holes is adhered to a Petri dish, which is then (**b**) exposed to air plasma to generate an array of hydrophilic disks. (**c**) The stencil is then removed, and the medium containing the bacteria is added to the hydrophilic disks, after which (**d**) the droplet array is covered with mineral oil.

Briefly, a μPLAT stencil is made from polydimethylsiloxane (PDMS) film, an inexpensive, soft polymer. The stencil has an array of 4 × 6 holes, each 3.18 mm in diameter, which is adhered to the surface of a Petri dish, (Figure 1a). The assembly is placed in a plasma cleaner (Harrick Plasma, Ithaca, NY, USA), and exposed to air plasma for 1 minute (Figure 1b). The stencil is removed after plasma treatment. Because only the polystyrene surface under the circular openings is exposed to the plasma, these areas become hydrophilic on the hydrophobic background of the plate (Figure 1c). The patterned MOCA Petri dish is then surface-sterilized under UV light for 15 minutes in a PCR cabinet (PCR Workstation; Thermo Fisher Scientific, Hudson, NH, USA). The bacteria and medium mixture is loaded using a pipette into the prepared Petri dish, producing a 4 × 6 array of 1-μl droplets. The borders of the liquid are confined at the hydrophilic/hydrophobic interface, forming discrete droplets defined by the stencil pattern. To prevent evaporation and contamination from the ambient environment, 5 ml mineral oil (M5904; Sigma Chemical Co., St Louis, MO, USA) is loaded to cover the droplet array (Figure 1d).

### Loading of bacteria in medium and culturing using microbe observation and cultivation array

Luria broth (Difco; Becton, Dickinson and Company, Sparks, MD, USA) was used for both bulk cell and droplet cultivation of the gram-negative bacterium *Escherichia coli* DH5α and the gram-positive bacterium *Bacillus subtilis* 168. Marine broth (Difco‘ Becton, Dickinson and Company) was used for droplet cultivation of marine bacteria from sea water sampled from the deep sea of the northwest Pacific Ocean [24]. Cells were counted using a counting chamber (Hausser Scientific Partnership, Horsham, PA, USA) under a microscope to estimate the bacterial density for both pure cultures and sampled sea water. Four cell concentrations (10^3^, 10^2^, 10, and 1 cell(s)/μl) were used in the experiments. Cell occupancy in the droplets is a Poisson random variable [23]; when 1 cell/μl medium is loaded into the 1 μl droplets, the average cell occupancy is one cell per droplet.

For each individual hydrophilic MOCA spots, 1 μl bacteria-medium mixture was pipetted onto it. The droplets were then covered with 5 ml of mineral oil and the lid placed on the Petri dish. The dishes were kept in the dark at room temperature, and growth was monitored by direct microscopy.

### Automated time-series observation

**Figure 2:**
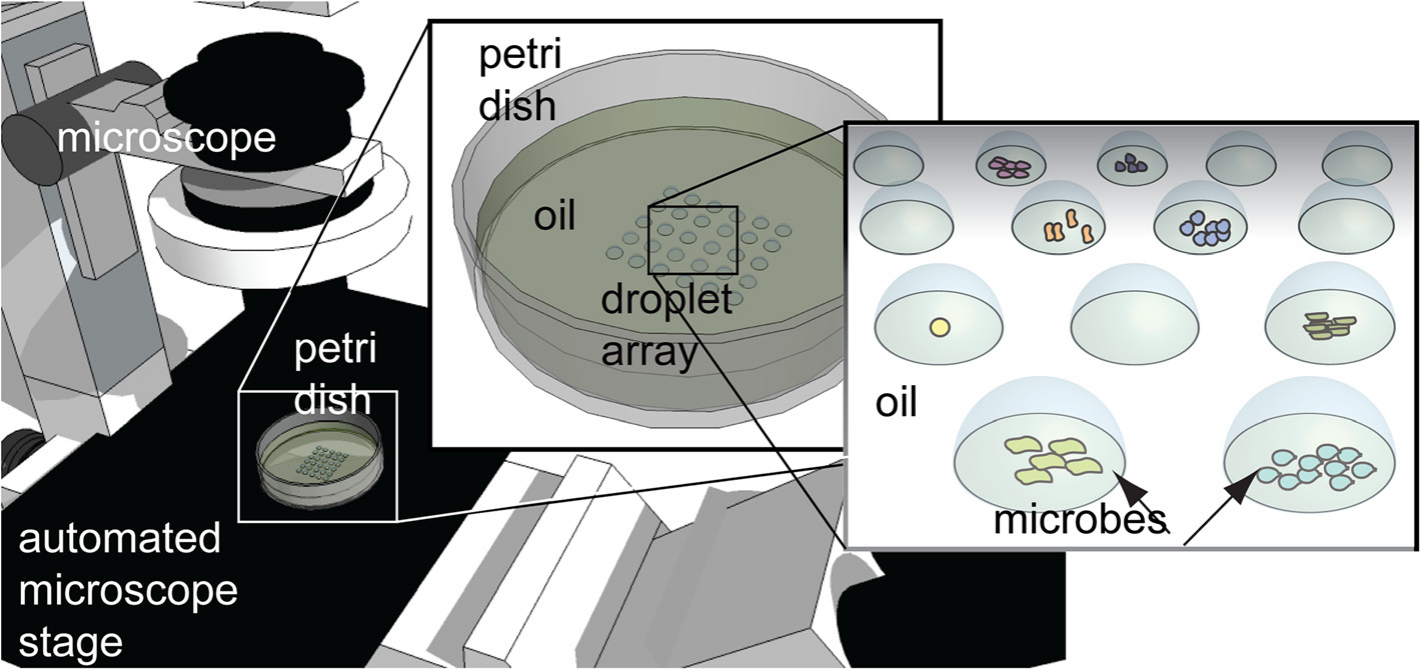
Demonstration of automated time-series observation by optical microscopy of bacterial droplet cultivation using microbe observation and cultivation array (MOCA).

A MOCA dish can be monitored on an automated optical microscope to observe the growth of the loaded bacteria. The MOCA Petri dish was placed on the automated microscope (Eclipse TiE, Nikon Instrument Inc., Japan) with a 4× objective, so the field of view covered an entire droplet (Figure 2). The microscope stage was programmed to travel through all the droplets and to acquire bright-field images of all droplets every 30 minutes for 72 hours. The microscope illumination was turned on only during image acquisition to avoid heating of the dish. After the experiments, the time-lapse micrographs of each droplet were used to estimate bacterial growth using measurement of light transmission; the micrographs appeared darker as bacteria grew. Although an automated microscope was used in this study, other optical platforms (for example, conventional microscopes or even the naked eye when the droplets are saturated with microbes) can also be used for estimation.

### Phylogeny analysis of isolated bacteria from single cells

The cultivated isolates developed from single bacterial cells of interest can be transferred using a micropipette from the MOCA chip to other containers for archival and downstream phylogenetic analyses using 16S rRNA sequencing. In this study, bacteria were removed from the droplets using a micropipette and transferred to a 1.5 ml microcentrifuge tube containing 100 μl sterilized water. After being spun in a centrifuge at 12000 *g* for 1 minute, the supernatant was decanted to remove possible carry-over of the mineral oil, then the bacterial cells were re-suspended in 5 μl sterilized water and transferred to a 0.2 ml PCR tube. The PCR tubes were then put in a PCR thermal cycler (model 9700; Applied Biosystems Inc., Foster City, CA, USA) and heated at 95°C for 5 minutes, followed by chilling on ice to induce cell lysis. Using heat-treated bacterial cells as the template, conventional PCR was carried out to amplify the highly variable V6 region of the small subunit (SSU) rRNA gene. The PCR primer sequences are shown in Table 1[25]. The 20 μl PCR volume contained: 10 μl 2 × PCR Master Mix (Fermentas Life Science Inc., Glenburnie, MD, USA), 2 μl of each primer (4 μmol/l), 2 μl of DNA template and 4 μl of double-distilled water. The conditions for PCR amplification were as follows: 94°C for 5 minutes; 35 cycles of 94°C for 15 seconds, 56°C for 20 seconds, and 72°C for 45 seconds; with a final extension step of 72°C for 10 minutes and soak at 4°C. The expected PCR products were recovered with a gel and extracted (Qiaquick Gel DNA Extraction Kit; Qiagen Inc., Valencia, CA, USA). The purified PCR products were used as templates for DNA sequencing, using a commercial kit (BigDye Terminator Cycle Sequencing Kit, version 3.1; Applied Biosystems Inc.) and a PCR analyzer (model 3700; Applied Biosystems Inc.) using the same PCR primers as before. The resultant DNA sequences were edited manually using Sequence Scanner Software (version 2.0; Applied Biosystems Inc.). Sequences were compared against those in GenBank through the National Center for Biotechnology Information (NCBI) portal and BLAST software (version 2.2.10) [26], and the phylogeny affiliation of each isolates were then inferred.

**Table 1:** Primers used for PCR

**Primer name**	**Sequence (5′→3′)**
U968	AACGCGAAGAACCTTAC-
L1401	CGGTGTGTACAAGACCC

## Results and discussion

### Bacterial growth in droplets

Because MOCA droplet settings create a different physical environment from that of bulk cultures, this may influence many of the growth factors required by the bacteria [27,28]. Thus, given that the total volume for cultivation is a microliter, bacterial growth within the droplets may have different properties from that in regular cultures. To test if the droplets could inhibit bacterial growth, we initially cultivated *E. coli* and *B. subtilis* cells within droplets with a cover of mineral oil and assessed their growth in real time. Using the method described above, a 4 × 6 array of 1 μl droplets for each species were generated for the four cell concentrations (10^3^, 10^2^, 10, 1 cell(s)/μl), using six droplets for each cell concentration. Cell occupancy in the droplets is a Poisson random variable [23]: when loading 1 cell/μl medium into droplets, around 37% of droplets are empty, and 37%, 18%, 6%, and 1.5% of the droplets are occupied by one, two, three, and four cells, respectively. Therefore, the lowest cell concentration in all presented cultivation experiments yielded single-digit bacterial cells in droplets.

**Figure 3:**
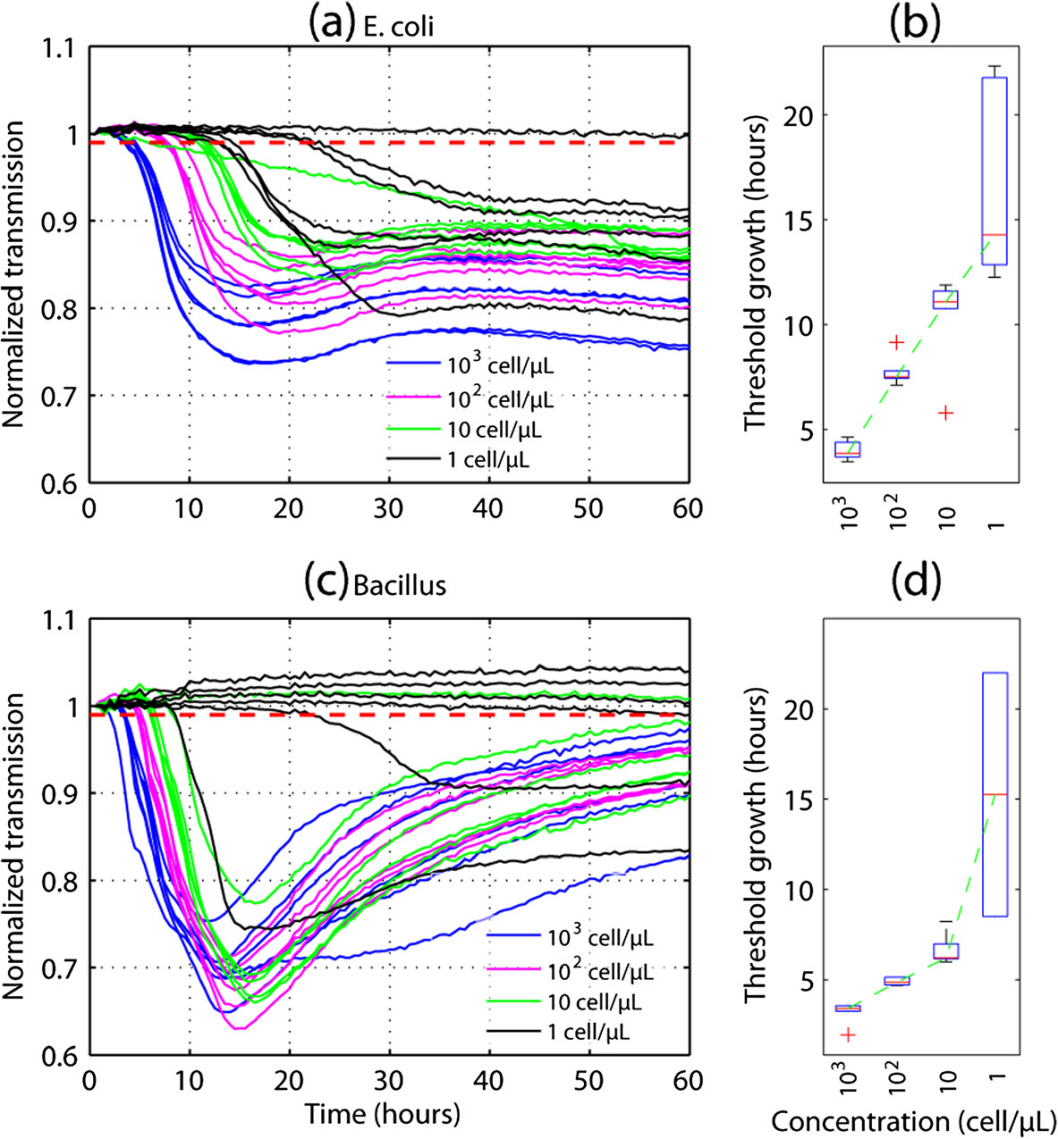
**Real-time growth record of bacteria in microbe observation and cultivation array (MOCA).** (**a,c**) Growth curves of (**a**) *Escherichia coli* and (**c**) *Bacillus subtilis* with different initial cell concentrations (the lower the transmission, the larger the cell population). Each curve represents the growth in an individual droplet. (**b,d**) Threshold growth time distribution versus (**b**) *E. coli* and (**d**) *B. subtilis* cell concentrations. The threshold growth time was defined as the time when the normalized transmission intensity of a droplet intersected 0.99 (Figure 3a and 3c, red dotted line).

Figure 3 shows the growth of these two bacteria, where each curve represents the growth in an individual droplet. We measured light transmission through the acquired micrographs as an indication of cell density; the lower the transmission, the larger the cell population. The transmitted light intensity was normalized with respect to its initial value to reduce the light variation between droplets., therefore, all growth curves started from 1. The colors of the curves in the figure indicate initial cell concentrations. The curves of the individual cell concentration were clustered before reaching the stationary phase, with a few outliers appearing for droplets with low initial cell concentrations. We defined the threshold growth time as the time when the normalized transmission intensity of a droplet intersected 0.99 (Figure 3, red dotted lines). The box plots (Figure 3b,d) show the threshold growth time, which was mostly linear to the individual cell concentrations. Because of random cell occupancy and cellular heterogeneity, the variation was large when bacterial number within the droplets was in single figures.

The growth curves of both *E. coli* (Figure 3a) and *B. subtilis* (Figure 3c) shared similar features. The curves of individual cell concentration were clustered before growth reached the stationary phase. One distinct feature of the *B. subtilis* growth is that the transmission intensity became brighter after the population peaked. The time-lapse micrographs showed that the number of cells did not significantly change, but the size of cells became smaller, suggesting that the *B. subtilis* cells started to spore after the nutrition was depleted [29]. Only two of the most diluted droplets contained *B. subtilis* cells that were able to grow into colonies in this experiment. Similar to the *E. coli* cultivation result, the threshold growth time was mostly linear to individual cell concentrations (Figure 3d), whereas the lowest concentration had a large variation because only two droplets resulted in growth.

These results show that, when the initial cell density is more than 10 cells/μl, bacterial growth across the droplet array is strongly uniform and reproducible. For droplets with single-cell occupancy, the platform can be used to monitor the growth heterogeneity at the single-cell resolution.

### Morphological development of microcolonies within droplets

**Figure 4:**
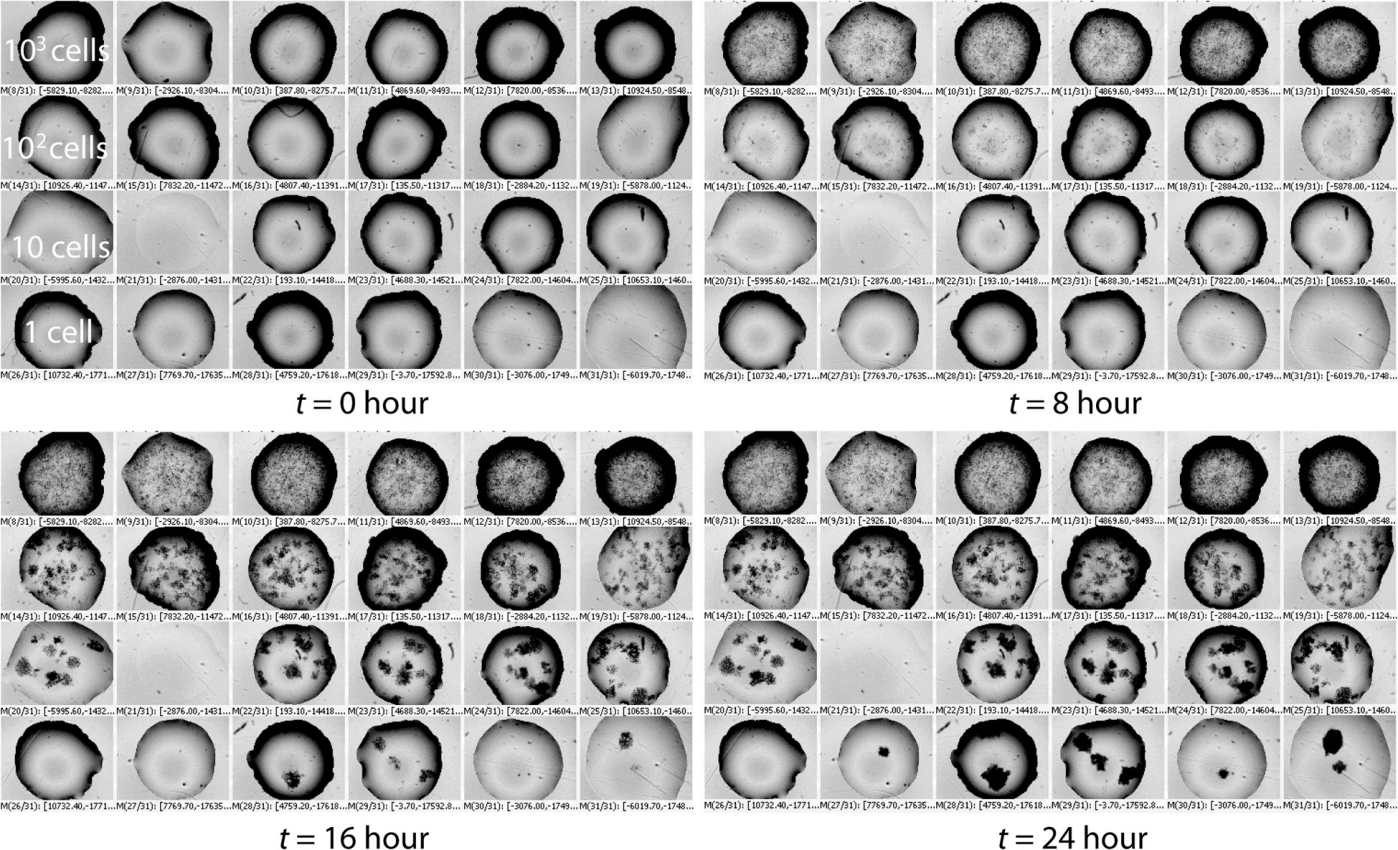
**Proliferation of *Escherichia coli *in droplets.** The time-series micrographs of 24 droplets at 0, 8, 16, and 24 hours. The dark clusters in the droplets are *E. coli* colonies. For all four panels, the nominal cell numbers of the five rows are 10^4^, 10^3^, 10^2^, 10, and 1 cell(s) per droplet, respectively.

We found that the droplet setting permitted the growth of both *E. coli* and *B. subtilis*. In addition, we also found that the bacterial growth pattern within a droplet was different from that of a regular liquid medium. This was especially evident when cultivating single-digit numbers of bacterial cells within a droplet. Using the time-series micrographs of the 24 droplets with *E. coli* growth recorded at 0, 8, 16, and 24 hours (Figure 4), we found that each single *E. coli* cell could develop its own microcolony, and these did not merge with each other until the solution became cloudy. Similar results were seen for *B. subtilis* (data not shown). In this respect, the growth pattern of both *E. coli* and *B. subtilis* within a droplet is more similar to that on a solid agar plate than in a liquid medium. This may due to the use of the oil droplet cultivation platform, in which the physical environment is different from that of routine liquid cultivation. For instance, oil coverage may create a microoxic environment, which may inhibit or reduce the movement of bacteria [30].

### Environmental bacteria cultivation and isolation using a microbe observation and cultivation array

We further used MOCA to cultivate and isolate environmental marine microbes, which were sampled from the Hydrate Ridge site (80 km off the Oregon coast and 5 m above the 780-m deep seafloor) during a research cruise between 22 July and 5 August 2008 [24]. To avoid loading multiple cells from each droplet, the cell concentration was diluted to 0.25 cells per droplet with marine broth medium. Based on Poisson statistics, the possibility of multicell occupancy with this concentration is around 2% and that of single-cell occupancy is 20%.

Using the automated time-series observation system, we could identify which droplets had bacterial growth and how many microcolonies had been developed within these droplets. Droplets showing single microcolony growth were identified and the cultivated pure microbes recovered directly. For verification of purity, the recovered cultures were subjected to further purification on traditional Petri dishes.

**Figure 5:**
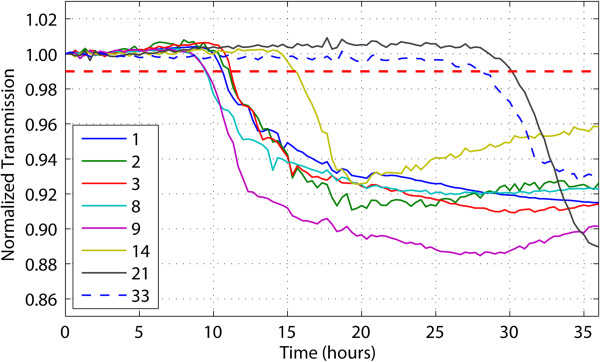
**Growth curve of marine bacterial isolates within the droplets.** Each curve represents the growth in an individual droplet. The number in the legend is the droplet number associated with Table 2.

In one experiment, thirty-six droplets were generated, and eight of these were successfully cultivated during a 2-day culture period. Time-lapse micrographs showed that they were all derived from a single bacterium (data not shown). The profiles of the growth curves showed similar trends to those of the previous results of the single-digit bacterial cell growth in a droplet (Figure 3), but the variation was larger, perhaps due to species differences or to the different time requirement for exit from dormancy status (Figure 5). Some fast-growing droplets (for example, those in droplets 8 and 9) started growing within the first 8 hours, whereas others (for example, droplet 21) started as late as the 30th hour. The threshold growth times of these droplets are listed in Table 2.

**Table 2:** Cultivated bacteria, their phylogeny affiliation and corresponding threshold growth times

**Droplet number**	**GenBank accession number**	**Closest match in GenBank (percentage identity)**	**Threshold growth time, hours**
1	JQ178347	*Pseudoalteromonas elyakovii* (99%)	10.51
2	JQ178348	*Pseudoalteromonas tetraodonis* (99%)	11.02
3	JQ178349	*Pseudoalteromonas tetraodonis* (99%)	11.16
8	JQ178350	*Pseudoalteromonas tetraodonis* (96%)	9.43
9	JQ178351	*Pseudoalteromonas tetraodonis* (98%)	9.48
14	JQ178352	*Pseudoalteromonas tetraodonis* (99%)	15.37
21	JQ178353	*Shewanella halifaxensis* (98%)	31.13
33	JQ178354	*Colwellia piezophila* (98%)	27.94

Single colonies were identified in these droplets and the transferred cells were subjected to colony PCR. After sequencing the obtained PCR products of 400 bp, which represent the highly variable V6 region of the SSU rRNA gene, a phylogeny analysis was conducted based on NCBI database searches. Sequences were deposited in GenBank under accession numbers JQ178347 to JQ178354. The closest species/genus with which each isolated strain was identified are listed in Table 2. Of these identified cultures, the majority (six out of eight cultures) belonged to *Pseudoalteromonas* spp. (E value = 0), with the remaining two identified as *Shewanella* sp. (E value = 0) and *Colwellia piezophila* (E value = 0), respectively. The phylogeny affiliation of recovered isolates is consistent with the source of the microbial community [31-33].

Because MOCA droplets create a different physical environment from that of bulk cultures, , this may influence many of the growth factors required by bacteria. For example, the use of an oil cover reduces gas exchange with the ambient environment, which may favor the growth of microaerobic bacteria, but not strict aerobic bacteria. In addition, putting MOCA into an anaerobic chamber and loading with a bacteria-media mixture under anaerobic conditions can allow an anaerobic environment to be created. We will explore the potential of this technique and its limitations in cultivating novel environmental bacteria in future studies.

## Conclusions

We have developed a new system to cultivate bacteria in a droplet array. Our results show that the set-up of droplet does not inhibit the growth of bacteria, as we found that both *E. coli* and *B. subtilis* cells could proliferate uniformly across the MOCA. We also found that the bacterial growth pattern within a droplet is more similar to that on a solid than in a liquid medium. Given its flexibility, ease of set-up, and sensitivity to growth of a single bacterial cell, we believe that this droplet platform could provide a cost-efficient way to cultivate and isolate novel bacteria from different environments, and had the potential for use in bioassay screening.

## Abbreviations

MOCA: Microbe observation and cultivation array; DNA: Deoxyribonucleic acid; rRNA: Ribosomal ribonucleic acid; SSU rRNA: Small subunit ribosomal RNA; PCR: Polymerase chain reaction; μPLAT: Microscale plasma activated templating; PDMS: Polydimethylsiloxane.

## Competing interests

The authors declare that they have no competing interests.

## Authors’ contributions

WG conceived of the study, participated in its design, carried out the phylogenetic studies, and was involved in the interpretation of all data. DN and JN carried out the preparation, cultivation processes, and phylogenetic studies. WZ conceived of the study, participated in its design, and was involved in the interpretation of all data. SC conceived of the study, participated in its design, and analyzed the results. DM conceived of the study. All authors read and approved the final manuscript and were involved in the interpretation of data.
